# The impact of high and low dose ionising radiation on the central nervous system

**DOI:** 10.1016/j.redox.2016.08.002

**Published:** 2016-08-10

**Authors:** Calina Betlazar, Ryan J. Middleton, Richard B. Banati, Guo-Jun Liu

**Affiliations:** a Bioanalytics group, Life Sciences, Australian Nuclear Science and Technology Organisation (ANSTO), New Illawarra Road, Lucas Heights, NSW 2234, Australia; b Discipline of Medical Imaging & Radiation Sciences, Faculty of Health Sciences, The University of Sydney, 75 East Street, Lidcombe, NSW 2141, Australia

**Keywords:** Ionizing radiation, Reactive oxygen species (ROS), Antioxidants, Neuroinflammation, Microglia, Translocator Protein (TSPO)

## Abstract

Responses of the central nervous system (CNS) to stressors and injuries, such as ionising radiation, are modulated by the concomitant responses of the brains innate immune effector cells, microglia. Exposure to high doses of ionising radiation in brain tissue leads to the expression and release of biochemical mediators of ‘neuroinflammation’, such as pro-inflammatory cytokines and reactive oxygen species (ROS), leading to tissue destruction. Contrastingly, low dose ionising radiation may reduce vulnerability to subsequent exposure of ionising radiation, largely through the stimulation of adaptive responses, such as antioxidant defences. These disparate responses may be reflective of non-linear differential microglial activation at low and high doses, manifesting as an anti-inflammatory or pro-inflammatory functional state. Biomarkers of pathology in the brain, such as the mitochondrial Translocator Protein 18 kDa (TSPO), have facilitated *in vivo* characterisation of microglial activation and ‘neuroinflammation’ in many pathological states of the CNS, though the exact function of TSPO in these responses remains elusive. Based on the known responsiveness of TSPO expression to a wide range of noxious stimuli, we discuss TSPO as a potential biomarker of radiation-induced effects.

## Highlights

• Ionising radiation can modulate responses of microglial cells in the CNS.
• High doses can induce ROS formation, oxidative stress and neuroinflammation.
• Low doses can mitigate tissue damage via antioxidant defences.
• TSPO as a potential biomarker and modulator of radiation induced effects in the CNS.
• Non-linear differential microglial activation to high and low doses is proposed.

## Graphical abstract

**Figure:**
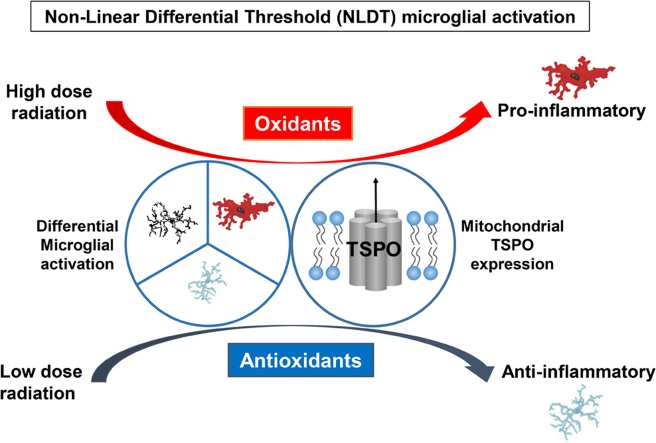

## 1 Introduction

The impact of ionising radiation on human physiology has been documented throughout the last century, subsequent to nuclear disasters and incidents through inhalation or ingestion of radioactive material [1]. Aside from environmental exposure, artificial sources of ionising radiation are growing in utility. Currently, exposure to ionising radiation through medical diagnostics and treatment strategies now constitutes the largest proportion of average yearly radiation exposure in Australia [2]. The increasing availability and utility of ionising radiation for medical purposes warrants a reassessment of the literature on the biological impact of higher and lower doses of ionising radiation, particularly in terms of the central nervous system (CNS). Whilst the neurobiological impact of exposure to high dose ionising radiation has been well documented, the consequences of low dose exposure have garnered considerable debate [3]. Responses of biological systems to ionising radiation are largely thought to follow a linear dose-response pattern [4]. Challenge to the prevailing paradigm of a linear no-threshold model comes in the form of animal radiobiological data, with several lines of evidence suggesting that exposure to lower doses of ionising radiation may confer neuroprotection [5], [6], [7], [8]. This response has been conceptualised as radiation hormesis, where exposure to a stressor in low amounts can induce protective, radioadaptive and reparative mechanisms [9], [10], though this is not without contention. Insufficient data expounding the beneficial effects of low dose ionising radiation on human physiology, and a lack of consensus regarding the definition of ‘low dose’, has meant that the concept of radiation hormesis is not currently acknowledged by international panels and governing bodies. Though there is still uncertainty surrounding the nature of biological responses to high and low dose ionising radiation, a more comprehensive understanding of the molecular and cellular processes underlying radiobiological responses is currently evolving [11], particularly within the context of the complex and multifaceted CNS.

Since its early discovery, radiation science has expanded in utility to medical practices [12], [13], [14], though it was not until later in history that the effects of ionising radiation on the brain were directly examined [15]. The paradox of utilising ionising radiation for therapeutic and medical diagnostic purposes is that at higher doses it may induce damage to normal tissue. The non-cancer effects of ionising radiation exposure, and the cellular reactions it can produce in the adult CNS, will be the focus of this review. In the literature, one of the more prominent manifestations of radiation-induced injury is seen in the hippocampus, a radiosensitive region housing populations of proliferating progenitor cells [16], [17], [18]. High dose irradiation can induce dysfunction or apoptosis to mature or newly born differentiating cells that integrate into the hippocampal network, manifesting as longer term functional deficits [19]. Orchestrating responses to high dose irradiation are microglial-mediated neuroinflammation and oxidative stress induced by excess reactive oxygen species (ROS) formation [20], [21]. Mitochondrial redox balance and microglial responses are also critical in modulating responses to low dose irradiation, largely through the stimulation of antioxidant defences [7]. Whilst some evidence still points to a linear dose-response pattern, there is significant evidence to suggest that lower doses can confer protection to cell functioning, molecular structures, synapses, and key brain mechanisms such as neurogenesis, and induce reparative mechanisms in the face of CNS pathology [10], [22]. Based on guidelines by regulatory bodies, as well as data generated by low dose radiation research programs, a low dose is considered to be acute exposure to less than 100mSv, or 0.1 Gy [23], [24]. Rather than adopting a stringent demarcation between ‘high’ and ‘low’ doses of ionising radiation, we will discuss ‘low dose’ data within the broader range of doses up to 1 Gy, a definition which extends across many radiobiological studies.

Here we review the literature regarding the impact of ionising radiation on the CNS, highlighting remaining uncertainties surrounding the disparate responses to high and low doses of ionising radiation that are underscored by mitochondrial redox balance and neuroinflammation. This review aims to synthesise the important aspects of CNS functionality under conditions of stress, injury and pathology, which can elucidate the neurobiological responses to ionising radiation at different doses. In order to enhance understanding in this field and dissect the subcellular and molecular events that drive radiation-induced neurobiological responses, a key biomarker of CNS pathology, the mitochondrial Translocator Protein 18 kDa (TSPO), will be examined. We introduce TSPO as a novel perspective in clarifying the responses of the CNS to ionising radiation, and highlight its centrality to a comprehensive understanding of the complex network linking neuroinflammation and mitochondrial redox balance. Its utility as a sensitive *in vivo* biomarker of microglial activation, coordinating the brains innate immune response, may lead to new insights into how this process may modulate CNS responses after high and low dose irradiation, as well as elucidating the exact function of this enigmatic protein.

## 2 Microglial responses to stressors in the CNS

The coordination of responses to insults and stressors in the CNS are complex and multifaceted. Neurobiological mechanisms of inflammation, protection, defence and repair comprise of networks of cells and molecular mediators that respond to alterations in homeostasis [25]. Neuroinflammation is inherent to an understanding of CNS responses after such alterations, for example exposure to ionising radiation. This mechanism is distinctively characterised by the presence of activated microglia, the brains innate immune effector cells, which exhibit striking morphological and functional plasticity in response to insults [26], [27]. In their resting state, microglia display highly ramified morphology and survey the microenvironment, though in the presence of endogenous or exogenous stressors, these cells can proliferate and transition morphologically to an amoeboid, activated state [28], [29], [30]. Activated microglia initiate an inflammatory response by releasing pro-inflammatory factors including cytokines and ROS [20], [31]. The pro-inflammatory state of activated microglia, or the M1 type classical activation state, can be cytotoxic to surrounding cells, and when unregulated can propagate tissue damage and cause secondary injury [32], [33]. Correspondingly, neuroinflammation is thought to be implicated in multifarious functions and pathological states in the CNS, including the modulation of neurogenesis and neuronal development [25], [34], [35], synaptic stripping and neuronal dysfunction [36], [37], and is now widely implicated in the pathogenesis and progression of many neurodegenerative disorders [38], [39], [40], [41], [42], [43], [44], [45]. Alternatively, an M2 microglial activation state is not neurotoxic, transiently conferring neuroprotection and anti-inflammatory properties in response to injury [46]. Microglial M2 activation and M2-derived factors have been demonstrated to promote remyelination and activate reparative and regenerative growth responses after lesions [47]. This activation state can also down-regulate inflammation, and reduces secondary injury which may be induced by inflammation [48], though the shift between M2 and M1 phenotypes is not well understood. The polarised activation states of microglia may also be implicated in the responses of the brain to ionising radiation, and furthermore, the disparate activation states may be reflective of the anti-inflammatory or pro-inflammatory responses of microglia after low and high dose ionising radiation, a topic which warrants future investigation.

Importantly, classically activated M1 microglia and its associated pro-inflammatory functions are intrinsically linked to the production of free radicals [20], [26], [49]. The production of ATP through oxidative phosphorylation results in the formation of oxidant by-products which can be damaging to cell components in sufficient quantities. The inability of antioxidant compounds and enzymes to neutralise the adverse effects of excess ROS contributes to oxidative stress, which can manifest as damage to nucleic acids, protein degradation, and lipid peroxidation in cells [50]. Concomitantly, reactive nitrogen species (RNS) and nitric oxide (NO) can also coordinate microglial responses to stressors in the CNS [51], [52], [53]. Activated microglia have also been shown to produce excess ROS and H_2_O_2_ from NADPH oxidases, which also generate large amounts of oxidants in activated phagocytic cells [54] and are important to the progression of ROS-mediated neuroinflammatory collateral damage to surrounding cell populations [55], [56]. Collectively, activated microglia can act through mechanisms such as the NF-κB pathway to release ROS, RNS and pro-inflammatory cytokines such as TNF-α, IL-1β and IL-6, or through MAPK signalling pathways that activate NADPH to coordinate neuroinflammation [20], [51], [57], [58], [59], [60]. Indeed, neuroinflammation and oxidative stress have been implicated in several CNS disorders, notably Alzheimer’s disease [61], [62] and Parkinson's Disease [40], [57], [63], potentiating cellular damage and pathology. The balance between oxidants and antioxidants is also fundamental to the normal physiological functioning of the CNS [64], as antioxidant processes have been shown to alleviate oxidative stress-induced damage and inflammatory responses by activated microglia [62], [65], [66]. Together, the aforementioned mechanisms are also critical in modulating responses of the irradiated CNS, and an understanding of the machinery behind neuroinflammation and redox balance can therefore help elucidate the responses of the CNS to ionising radiation at different doses.

## 3 The impact of high dose ionising radiation on the CNS

One of the earliest physiological responses to ionising radiation is the production of ROS [67], which can induce oxidative stress and neuroinflammatory processes at sufficiently high doses of ionising radiation. A recent meta-analysis of published data suggests an overall significant effect of oxidative damage in response to ionising radiation, particularly on cells of the brain. Interestingly, there was significant heterogeneity in effect sizes among species and cell types across the body [68], highlighting the large variability of responses to ionising radiation. Indeed, even within the CNS, the complex interrelationships between neuroinflammatory responses, mitochondrial redox mechanisms and different cell populations complicate the understanding of responses to ionising radiation at different doses [24]. It is clear, however, that ionising radiation can impact nucleic acids [69], [70], neurovascular permeability [71], [72], [73], induce ROS/RNS [21], [67], [74], [75] and elicit a neuroinflammatory response [76], [77], [78], all of which can manifest as radiation-induced injury.

The presence of cognitive decline in patients after clinical exposure to high dose irradiation has led to a research focus on the hippocampal microenvironment and its population of mature cells and proliferating progenitor cells in the subgranular zone of the dentate gyrus. This region of neurogenesis is particularly sensitive to ionising radiation [79], as mitotic cells are radiosensitive at stages of the cell cycle between the G2 and M phase [80], [81], [82], [83]. Doses of ionising radiation above 1 Gy can influence neural progenitor cell survival in the hippocampus by significantly reducing the number of newly differentiated cells, increasing DNA damage (γ-H2AX nuclear foci), promoting cell-cycle arrest, and inducing oxidative/nitrosative stress, which ultimately implicates the functionality of the hippocampus manifesting as cognitive impairment [84], [85], [86], [87], [88]. Cell viability and apoptosis are important implications of high dose irradiation across the cell populations of the CNS, as gamma irradiation at 10 Gy in cell cultures reduces cell survival and increases apoptosis through the up-regulation of key apoptotic proteins, including cytochrome-c, caspase-3 and decreased expression of anti-apoptotic Bcl-2. Concomitantly, elevated pro-inflammatory cytokines TNF-α, IL-1β and IL-6 and lipid peroxidation markers relative to sham irradiated controls also highlights critical inflammatory responses after high dose ionising radiation that can propagate radiation-induced injury [89].

**Fig. 1:**
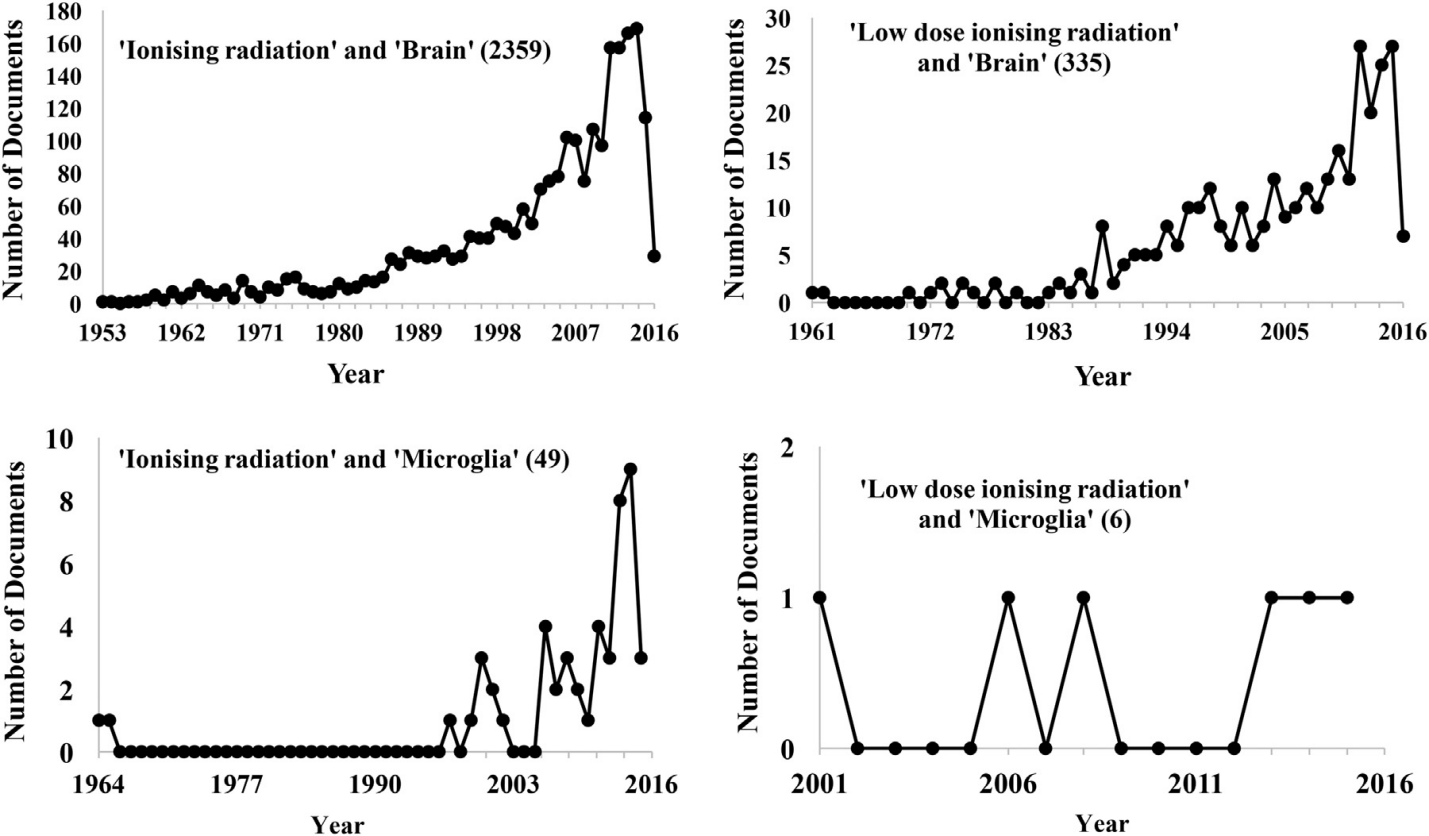
Publications trends using the SCOPUS database. ‘Ionising radiation’ and ‘microglia’ yields fewer results (49) than ‘ionising radiation’ and ‘brain’ search terms (2330). The lack of research between 1964 and 1997 is significant, and the striking increase in literature in the last 2 decades may coincide with the emergence of the concept of ‘neuroinflammation’ as a prolific research field within neuroscience. Even fewer results are yielded for ‘low dose ionising radiation’ (328), and fewer yet when ‘microglia’ is included (6), representing the paucity of studies on this topic. The second wave of publications on ‘low dose ionising radiation’ and ‘brain’ starting at the beginning of the 1970s may be as a result of the heightened use of medical radiation technologies and the consequent clinical exposure of patients to low doses.

**Fig. 2:**
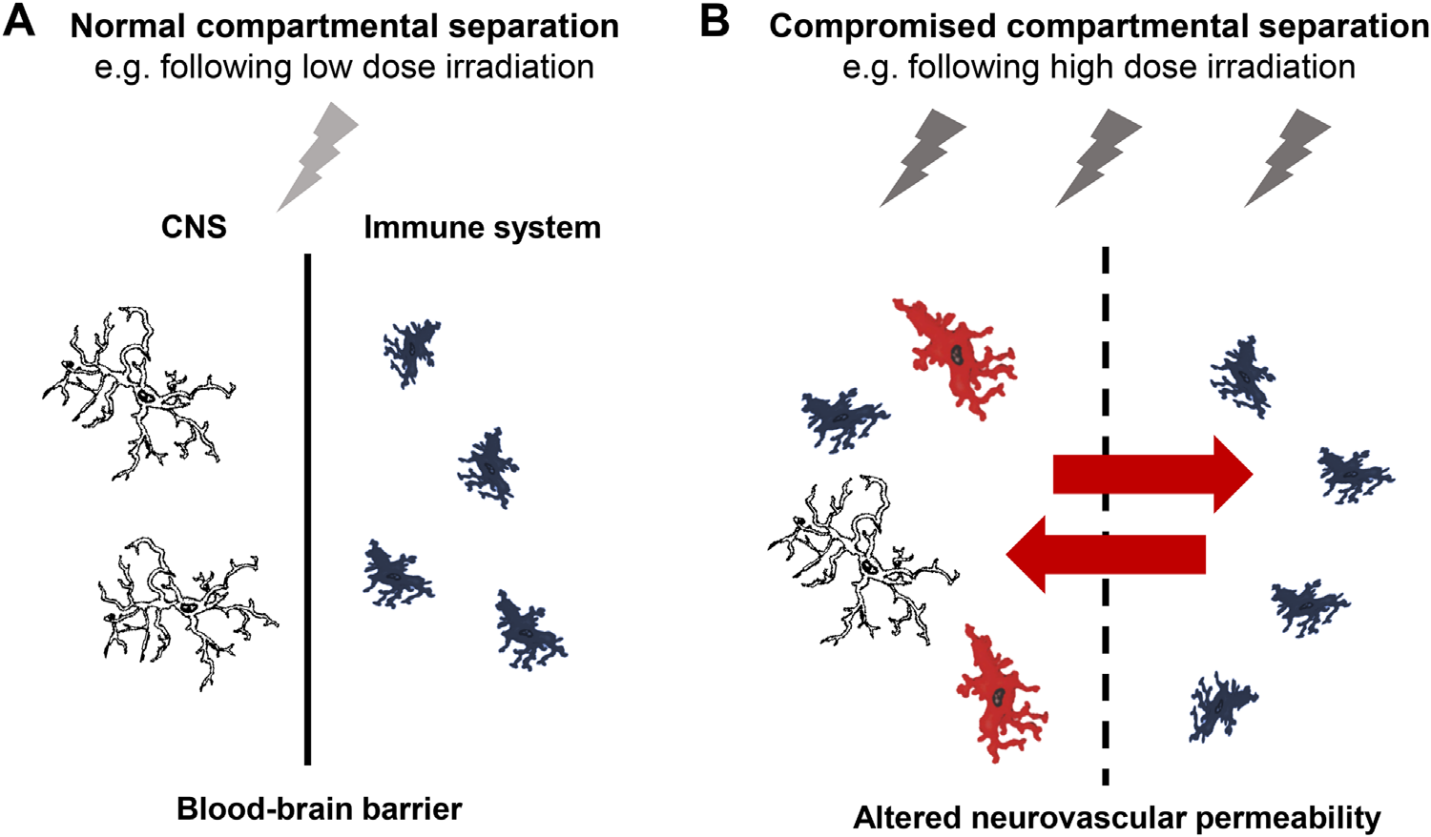
Radiation exposure compromises the integrity of the blood-brain barrier (BBB). Adapted from [146], [147]. (A) Normal compartmental separation of the CNS from the peripheral immune system represents an intact BBB. Compartmental separation may not be compromised following low dose radiation exposure. (B) High dose irradiation can induce damage and apoptosis of endothelial cells, resulting in the infiltration of peripheral macrophages (as indicated by red arrows), disturbing the normal compartmental separation between the CNS and the peripheral immune system. High doses can also induce microglial activation (cells in red), responsible for the brains innate immune response.

A search using the SCOPUS database for ‘ionising radiation’ and ‘microglia’ yields a steady increase in publication numbers since the earliest recorded data by Estable-Puig et al. in 1964 (refer to Fig. 1). Such was the first demonstration of cortical microglial activation, as well as the degeneration of myelin, in response to high dose particle irradiation [15]. It is now recognised that sufficient doses of ionising radiation can induce microglial activation and pro-inflammatory derived factors which can propagate radiation-induced brain injury [90], [91]. Correspondingly, radiation-induced neuroinflammation can inhibit adult neurogenesis in the hippocampus [92], [93], [94], [95], with longer term cognitive deficits in patients persisting from months to decades after initial exposure [96]. Interestingly, the finding of reversible inhibition of the proliferative capacity of progenitor cells in the dentate gyrus 1 week after 4 Gy X-radiation exposure, with an absence of microglial activation [97], suggests there is a requirement of neuroinflammatory processes to cause persistent and deleterious effects to cells [98]. In the important work of Monje et al. (2003), 10 Gy X-irradiation in adult mice incites microglial activation in the hippocampus, causing significant reductions in the proliferative capacity of progenitor cells, impacting cell fate. This neuroinflammatory response was alleviated during and after irradiation with application of an anti-inflammatory agent, partially restoring the proliferative capacity of these cells [98]. Activated microglia can inhibit neurogenesis after higher doses of ionising radiation through the release of pro-inflammatory cytokines IL-1β, TNF-α and IL-6, also contributing to progenitor cell dysfunction [84], [98], [99], [100], [101]. Importantly, the pro-inflammatory reactions of microglia subsequent to high dose irradiation typify the M1 microglial activation state, as opposed to M2 phenotypye [102]. The pro-inflammatory microenvironment after irradiation may also develop as a result of disruption to the blood brain barrier [103], [104] (as illustrated in Fig. 2). Doses above 5 Gy can promote apoptosis of endothelial cells in the brain, thereby causing microvascular damage, as well as contributing to radiation-induced cognitive dysfunction [105]. Compromise of the blood brain barrier can result in increases of peripherally derived macrophages [99], or brain infiltrating leukocytes, expressing factors such as CCR2 or ICAM-1 [106]. Radiation also increases vulnerability of the brains microvasculature and perivascular cells, which can lead to increased DNA damage and premature senescence in surviving cells [73].

The induction of pro-inflammatory factors can occur through pronounced activation of transcription factors such as NF-κb, causing aberrant regulation of genes involved in pathological inflammatory states [91], [107], [108], and can also induce apoptosis [67], [109]. Correspondingly, Schnegg et al. [108] applied the anti-inflammatory and antioxidant PPARδ agonist to BV-2 microglial cell cultures prior to a single dose of 10 Gy gamma radiation. This agent alleviated radiation-induced neuroinflammation by reducing TNF-α, IL-1β and COX-2 expression via NF-κB, whilst also inhibiting ROS generation, revealing the critical importance of ROS modulation in neuroinflammatory responses after high dose ionising radiation. PPARδ activation also inhibited phosphorylation of c-Jun and MEK/ERK1/2 which regulated the inhibition of ROS formation and the expression of pro-inflammatory factors [108]. A congruous effect has also been demonstrated utilising other classes of PPAR agonists, adding to the significance of these mechanisms in the reactions of microglia following high dose irradiation [110]. Analogously, Deng et al. (2012) demonstrated the importance of the MAPK MEK/ERK1/2 signalling cascade in mediating the responses of microglia after high dose irradiation. Exposure of BV-2 microglial cell cultures to 10 Gy gamma radiation induced the phosphorylation of c-Jun and ERK1/2, which correlated with increases in the expression of pro-inflammatory factors and ROS. Application of NADPH oxidase inhibitors resulted in a marked reduction in ROS after irradiation and decreased c-Jun phosphorylation, indicating that radiation-induced oxidative stress stimulates MEK/ERK1/2 signalling cascade to activate c-Jun, inciting a neuroinflammatory response in microglial cells [49].

Oxidative stress and redox balance play critical roles in the responses of cells to higher doses of ionising radiation [21], [95], [111]. Acute increases in mitochondrial ROS/RNS generation at a dose-dependent rate from 1 to 10 Gy, accompanied by changes in mitochondrial membrane potential, and mitochondrial permeability, characterise one of the most widely observed cellular reactions to ionising radiation [112]. Acute increases in lipid peroxidation after exposure to doses above 2 Gy, measured as a function of malondialdehyde (MDA) levels in brain tissue, can persist months after exposure [113], signifying sustained oxidative stress-induced injury. Accompanying increases in oxidative stress are increases in deleterious DNA damage such as double strand breaks (DSBs) and decreases in DNA repair proteins, which in turn can lead to apoptosis via cell-cycle arrest pathways and even reductions in cortical thickness [114]. Oxidative stress mediated damage in the brain can also occur as a result of diminished antioxidant enzyme activity such as superoxide dismutases (SOD), glutathione and catalase after irradiation above 2 Gy, which typically counteract the deleterious effects of ROS accumulation in cells [115]. The importance of antioxidant defences is highlighted by the finding of linear decreases from 2 to 5 Gy in the activity of Cu/Zn/Mn-SOD in both the hippocampus and the cortex up to 24 h post-exposure. The hippocampus displayed more significant decreases in antioxidant activity, highlighting the greater sensitivity and impaired recovery capacity of this region [80]. More recently, Ismail et al. [113] demonstrated that after a single dose of 6 Gy gamma radiation in rodents, there were significant increases in DNA DSBs, increased levels of lipid peroxidation, with a concomitant reduction in glutathione and SOD activity compared to sham irradiated controls. These responses were ameliorated in the group pre-treated with an antioxidant agent, demonstrating the importance of antioxidative neutralisation of oxidative stress-induced injury after high dose irradiation [113]. Interestingly, the integral importance of antioxidants has been highlighted in studies utilising mitochondrial catalase overexpression. This overexpression neutralises lipid peroxidation in the hippocampal region after exposure to doses above 2 Gy [116], as well as preserving neuronal and dendritic networks in the hippocampus, ultimately decreasing radiation-induced cognitive dysfunction [117].

Though there are inconsistencies in the dosages, dose rates, radiation sources (refer to Table 1) and *in vitro* and *in vivo* methodologies used in studies, it is apparent that responses of the CNS to higher doses of ionising radiation are underscored by excess ROS production, contributing to oxidative stress related damage and neuroinflammation. It is interesting to note that many of the studies examined here, and indeed throughout the literature in general, apply a single dose irradiation to tissue and examine the effects thereafter (as highlighted in Table 1), which may not be predictive of outcomes after other dosing schedules. Greene-Schloesser et al. (2010) demonstrated that in the hippocampal dentate gyrus, there was greater activation and proliferation of microglia after single dose exposure compared to corresponding fractionated doses delivered in 5 Gy fractions twice a week for 2–4 weeks. This serves to highlight the differences in cell responses to dose fractions as compared with administration of a single acute dose [118], and in particular, may highlight the sensitivity of microglial reactivity to the acuteness of the stressor that is delivered to the brain.

## 4 The impact of low dose ionising radiation on the CNS

A large amount of uncertainty remains concerning the impact of low dose ionising radiation on the CNS. Additionally, there is a lack of consensus regarding the definitions of ‘low dose’ ionising radiation, in terms of both total dose and dose rates. An examination of publication trends reveals a relative decrease in the literature regarding low dose ionising radiation and the brain as compared to higher doses, emphasising the lack of uncertainty surrounding the neurobiological effects of low doses (see Fig. 1). Of the earliest research into acute low dose effects on the adult brain, Yamaoka et al. [119] provided demonstrable evidence of the activation of protective mechanisms in the brain, through the induction of antioxidant activity that mitigates lipid peroxides [119]. Though there is still contention surrounding the nature of responses to low dose irradiation, a prevalent theme in much of the literature indicates that low dose irradiation may not induce deleterious alterations in cognition, cell functioning, DNA and gene expression, apoptosis, nor pathological signs *in vivo* [120], and that it may in fact stimulate molecular and cellular protective mechanisms [8].

Moreover, where higher doses may incite a neuroinflammatory response, lower doses of gamma radiation at 0.5 Gy have been shown to attenuate inflammatory responses by suppressing the release of pro-inflammatory cytokines. Interestingly, the striking lack of publications regarding ‘low dose ionising radiation’ and ‘microglia’ from the SCOPUS database (refer to Fig. 1) highlights the relative quiescence of data on this topic. Though there is a paucity of literature directly measuring the responses of microglia to low dose irradiation, there is evidence of decreased neuroinflammation with concomitant suppression of ROS-mediated damage and apoptosis. Low dose irradiation of hippocampal neurons at 0.2 Gy has been demonstrated to enhance cell viability through the suppression of ROS 5 days post-exposure, compared to higher doses of 2 Gy which compromised cell survival [121]. Similarly, human neural stem cell cultures irradiated with charged particles at 0.05–0.25 Gy showed increased levels of ATP, and decreased ROS/RNS levels, which contributed to increased cell survival, as opposed to cells irradiated at higher doses of 1 Gy [122]. Thus, low dose ionising radiation may stimulate defences against neuroinflammation and attenuate oxidative stress, which crucially influences cell proliferation, cell functioning and ultimately cell survival in the CNS. Radioadaptive dosing, where cells are pre-primed with a low dose, can reduce vulnerability to subsequent exposure to higher doses, also producing a neuroprotective effect. Otsuka et al. [123] demonstrated that animals primed with pre-exposure to doses of 0.5 Gy gamma radiation developed increased resistance to DNA damage after subsequent exposure to a higher challenge dose of 1.6 Gy, compared to mice irradiated with the higher dose alone [123], indicative of a radioadaptive response [22], [124]. In terms of inflammation, decreases in pro-inflammatory markers in the mouse hippocampus and cortex were present only in animals primed with a 0.1 Gy dose prior to subsequent exposure to 2 Gy, suggesting that early exposure to low doses can prevent the upregulation of inflammation by higher doses [100]. Though radioadaptive responses have been demonstrated in different cell types, further exploration into the radioadaptive dosing paradigm in terms of its effects on the brains innate immune system is needed.

Importantly, evidence suggests that the responses of DNA and gene expression in the CNS after low doses of ionising radiation are qualitatively different from higher dose exposures. Important work from Yin et al. [125] demonstrated that 0.1 Gy gamma radiation can induce alterations in gene expression involved in neuroprotective functions, notably DNA repair, cell-cycle control, lipid metabolism and stress responses. Interestingly, late changes implicated genes involved in metabolic functions, myelin and protein synthesis and increases in transcripts for antioxidative enzymes [125]. Analysis of transcriptome profiles after low dose irradiation of 0.1 Gy has also demonstrated the acute alterations in expression levels of genes involved in damage responses, signalling pathways associated with cognition, and the downregulation of ERK/MAPK, which are significantly different to pathways induced by 2 Gy [126]. The importance of dose delivery schedules must also be highlighted, as protracted exposure to 0.05 Gy X-rays over 10 days alters molecular signalling pathways in the hippocampus and cortex, such as the expression and phosphorylation of protein kinases ERK1/2 and AKT, as well as decreasing hippocampal cell proliferation. These alterations were not present after administration of a corresponding single dose of 0.5 Gy [127], demonstrating that continuous exposure resulted in a more pronounced, deleterious effect. The striking incongruence in results as a function of altering the dose delivery method thus limits the ability of single acute doses to predict outcomes to continuous or protracted exposure.

In rodent models of neuropathology, low dose ionising radiation exposure has been shown to confer neuroprotection and activate reparative mechanisms. Kojima et al. (1998) utilised a 1-methyl-4-phenyl-1,2,3,6-tetrahydropyridine (MPTP) model of Parkinson’s Disease to demonstrate acute increases in the levels of glutathione and catalase 3 h after exposure to a single dose of 0.5 Gy gamma radiation, with concomitant neutralisation of lipid peroxidation [128]. This has been confirmed more recently in a mouse model of Parkinson’s Disease after whole body gamma radiation at 1.5 Gy, where irradiated diseased animals had demonstrable restoration of glutathione levels, and even elevated levels of striatal dopamine [129]. Such neuroprotective effects after exposure to the slightly higher dose of 1.5 Gy also serves to highlight the ambiguity surrounding the exact parameters of high and low doses. Higher doses of ionising radiation were once used therapeutically for lymphoid radiation to treat Multiple Sclerosis, though not without contention [130], [131], [132]. More recently, in experimental models of Multiple Sclerosis, low dose gamma radiation delivered at repeated doses of 0.5 Gy attenuated experimental autoimmune encephalomyelitis (EAE) though suppression of pro-inflammatory factors such as TNF-α, IL-6, cytotoxic T cells, and induced recruitment of regulatory T-cells, which are involved in the therapeutic effect [133], [134]. This anti-inflammatory effect has also been demonstrated in genetic autoimmune disease models in mice, where after 5 weeks of continuous 0.35 mGy/h and 1.2 mGy/h gamma irradiation, treated mice had attenuated cerebral inflammation, and increases in lifespan comparative to non-irradiated controls, indicating a persistent protective effect of low dose irradiation [135]. In animal models of brain injury and tissue damage, radon inhalation at 2000 Bq/m^3^ for 24 h mitigated neuronal injury in the hippocampus whilst also stimulating acute increases in the activity of SOD and glutathione levels compared to controls [136]. Comparable responses have been demonstrated after single dose 0.5 Gy X-irradiation, which can also elevate antioxidant levels in the presence of lesions, rescuing cells from apoptosis, relative to sham irradiated controls [137]. Interestingly, Otani et al. [138] demonstrated analogous findings in a neurodegenerative model of retinitis pigmentosa, where cranial exposure to 0.65 Gy gamma radiation in diseased mice rescued photoreceptor cells from apoptosis, increased cell survival, and prevented further degeneration compared to mice exposed to 2 Gy. Remarkably, *Prdx2* antioxidative gene upregulation was observed after this low dose exposure, with silencing of this gene causing the rescue effect to be lost [138].

Antioxidative mechanisms are evidently implicated in the neuroprotective and reparative responses that low dose ionising radiation can confer [139]. Interestingly, in studies of medical professionals and hospital staff, blood antioxidant levels were significantly higher in participants exposed to low dose ionising radiation ranging from 0.1 to 3.8 mGy per month compared to unexposed controls [140], [141], though the paucity of literature on human studies necessitates further investigation of this effect. In mice, whole body exposure to both 0.1 Gy and 1 Gy gamma radiation boosts levels of glutathione, catalase and SOD activity in brain tissue after 2 weeks, but returns to baseline at 4 weeks [142]. Corroborating evidence from Veeraraghavan et al. [143] demonstrated that after single dose exposures to 0.1 or 0.5 Gy gamma radiation in mice, increases in the gene expression of *SOD2* and SOD activity facilitated through the NF-κB and SOD signalling network persisted 8 days after exposure. Notably, antioxidant upregulation was not present after exposure to 0.02 Gy, suggesting that the mitochondrial machinery behind this response requires sufficient exogenous stimulation by low dose ionising radiation [143]. More recent evidence from Abdel-Rafei et al. [144] highlighting the importance of mitochondrial redox balance to low dose responses demonstrates that exposure to fractionated 0.5 Gy (twice 0.25 Gy at 2 d interval) gamma irradiation prior to biochemical insult can ameliorate lipid peroxidation and produce an anti-apoptotic effect in the hippocampus, whilst also increasing levels of glutathione, SOD and catalase activity in this region. Importantly, such responses also manifested as significant improvements in cognitive functioning [144].

There is a growing body of evidence to suggest that low dose ionising radiation exposure to the CNS produces responses that are consistent with the radiation hormesis model, though inconsistencies in the literature emphasise the need for further research in this field. Recently, Katsura et al. [145] irradiated human neural progenitor cell cultures with low dose gamma radiation at 3 very low doses rates corresponding to 0.031–0.496 Gy delivered continuously for 72 hours. Demonstrable dose-dependent alterations in DNA DSBs, increases in inflammatory markers, cell-cycle arrest and increased gene expression of apoptotic pathways ultimately impacted cell differentiation and survival [145]. These findings emphasise the importance of dose rate as well as total dose (refer to Table 2), schedule of delivery, and *in vivo* and *in vitro* methodology, all of which are varied across the majority of studies. Dose fractionation, protraction or acute single dose delivery can all manifest different neurobiological outcomes, from the molecular to the cellular level, which needs to be considered in future studies. Despite the heterogeneity in experimental conditions throughout the literature, it is clear that a salient trend across the radiobiological data points to a protective and adaptive effect of low dose ionising radiation. In order to further characterise CNS responses to low dose irradiation, and address the remaining uncertainties, a direct examination of microglial responses, as well as the machinery driving antioxidant stimulation, will need to be determined. The potential induction of an M2 microglial activation state following low dose irradiation, which can down-regulate inflammatory responses and induce reparative mechanisms in response to injury, should also be explored in future studies. This may also delineate the phenotypes of microglia in response to varying stimuli in the CNS.

## 5 Mitochondrial Translocator Protein 18 kDa (TSPO) in neuroinflammation and responses to ionising radiation

An understanding of the mitochondrial mechanisms that drive oxidant/antioxidant production and microglial activation after irradiation remains elusive. Given the remaining uncertainties surrounding the susceptibility of the CNS to ionising radiation at different doses, new research modalities and approaches to the study of mitochondrial biology are needed. Enhanced understanding of the interdependence between mitochondrial mechanisms and neuroinflammation has been facilitated through sensitive *in vivo* neuroimaging markers, such as the mitochondrial Translocator Protein 18 kDa (TSPO), which has garnered considerable attention [148], [149]. Though its exact role in cellular functioning remains elusive, TSPO has been regarded as integral to cholesterol transport and steroid hormone synthesis, consistent with its nomenclature [150]. However, the creation of a viable TSPO knockout mouse model by Banati et al. [151] has challenged this function, indicating that the role of TSPO lies beyond steroidogenesis and cholesterol translocation [151], [152]. The TSPO knockout mouse model will facilitate further studies into the role of this outer mitochondrial membrane protein in cellular functioning [152], where it is hoped that further studies will provide a novel perspective for TSPO in responses after ionising radiation.

Interestingly, in the CNS, TSPO expression is quiescent under normal physiological conditions, yet is predominantly, if not exclusively, upregulated in activated microglia under conditions of stress or pathology [153], [154], [155], [156]. This has spurned prolific investigations into its utility as a biomarker of neuroinflammation [146], [157]. It should be noted that perivascular cells and endothelial cells too express binding sites for TSPO ligands [158], though the TSPO regulation in this compartment has not yet been fully investigated. Specific ligands and the use of *in vivo* neuroimaging techniques such as Positron Emission Tomography (PET) have implicated TSPO in multifarious disease states of the nervous system, including neurodegenerative disorders [159], [160], [161], [162], dementia [163], amyotrophic lateral sclerosis [164], [165], multiple sclerosis [158], [166], [167] and brain tumours [168], [169]. In experimental models, ligands of TSPO such as PK11195 have been found to reduce pro-inflammatory gene expression of COX-2, TNF-α and IL-6 after stimulation with lipopolysaccharide (LPS) [170], [171], with a concomitant reduction in the number of activated microglia, sparing further degeneration [172]. Thus, TSPO is now the target of research into high affinity therapeutic ligands against various pathologies of the CNS [173].

Functional characterisation of this protein within mitochondria remains elusive. Furthermore, the mechanisms by which TSPO contributes to microglial activation remains a topic of great interest, and enhanced understanding may be useful for uncovering novel insights into responses after ionising radiation exposure. Currently, TSPO deletion in mice has uncovered the role of TSPO in mitochondrial bioenergetics, as TSPO deficient mice display significant decreases in oxygen consumption [174], and produce significantly less ATP in microglial cell cultures compared to wildtype mice [151]. As the production of mitochondrial ROS is intrinsically linked to ATP production, TSPO has also been implicated in the regulation and production of ROS, and ultimately, ROS-mediated oxidative damage and apoptosis [175]. Veenman et al. (2010) demonstrated that activation of TSPO facilitates ROS generation and ATP depletion, with involvement of the mitochondrial F_o_F_1_-ATP(synth)ase, leading to the activation of caspase-3, collapse of mitochondrial membrane potential, and the induction of apoptosis [176], [177]. Indeed, several lines of evidence demonstrate an intrinsic role of TSPO in ROS generation, which ultimately impacts cell viability and survival. TSPO can up-regulate the production of ROS in complex with the voltage-dependent anion channel (VDAC1) of the outer mitochondrial membrane, as overexpression of the protein is associated with increased oxidative stress [178] and involvement in apoptotic pathways [179]. Lin et al. [180] demonstrated that inactivation of the *Tspo* homolog in Drosophila decreased caspase 3/7 activity and inhibited apoptosis triggered by 30 Gy gamma radiation and H_2_O_2_ exposure. Moreover, Aβ42-induced neurodegeneration was mitigated after *Tspo* depletion and male lifespan increased in diseased flies, providing novel evidence of the role of TSPO after radiation exposure and oxidative stress [180].

Other putative links between TSPO and ROS formation involve protoporphyrin IX (PPIX), which promotes cell death through ROS formation in complex with TSPO [181]. Furthermore, activation of TSPO by NO is associated with apoptosis in glioblastoma cell lines via collapse of mitochondrial membrane potential, mitochondrial ROS formation and DNA fragmentation, revealing a link between TSPO-NO cytotoxicity [182]. Santoro et al. [183] recently demonstrated that application of synthetic TSPO ligands from the N,N-dialkyl-2-phenylindol-3-ylglyoxylamides class to C6 glioma cell line cultures correlates with reduced lipid peroxidation and reductions in iNOS and COX-2 expression in LPS-stimulated cells [183], providing further evidence of the involvement of TSPO in the induction of pro-inflammatory factors and ROS-mediated oxidative stress. Interestingly, Choi et al. [184] found that exposure of microglial cell cultures to prototypical TSPO ligands PK11195 and Ro5–4864 increased microglial proliferation, phagocytosis, ROS production and cytokine release relative to vehicle controls. Importantly, ROS production was mitigated with application of NADPH oxidase inhibitors, suggesting an interaction between TSPO and NADPH oxidase in the production of ROS in microglia [54], [184]. In microglia, NADPH oxidase is modulated by JNK signalling cascade which contributes to the production of ROS [60], and importantly this has also been demonstrated subsequent to 10 Gy radiation exposure in microglial cell cultures [49]. Interestingly, TSPO gene and protein expression may be also driven by signalling pathways involving MAPK through AP-1 transcription factors [185], revealing a putative connection between TSPO and ROS formation that may be implicated after exposure to ionising radiation.

**Fig. 3:**
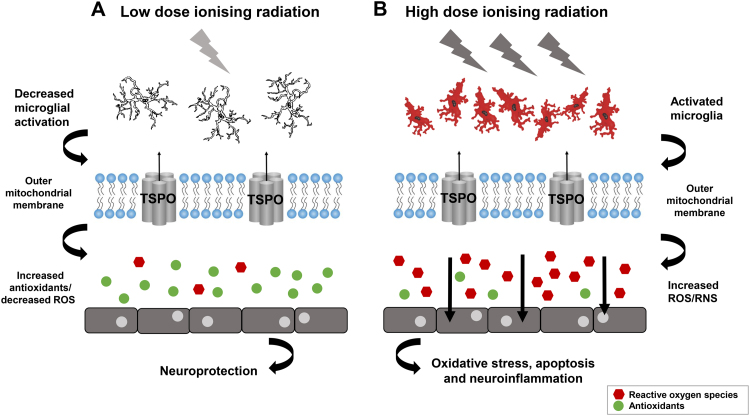
Ionising radiation and the cellular and molecular mediators of responses in the CNS. (A) Low dose ionising radiation may confer neuroprotection by decreasing neuroinflammation, increasing antioxidant levels and neutralising oxidative stress. (B) High dose ionising radiation provokes a neuroinflammatory response via activated microglia (cells in red), pro-inflammatory cytokines and reactive oxygen species (ROS) which can have deleterious effects on cell functioning and survival. The role of TSPO in modulating ROS may also implicate this protein in redox balance after ionising radiation at different doses. Adapted from [146], [147].

Further research directed in this field may lead to novel insights into the postulated link between TPSO, ROS and neuroinflammation in the coordination of responses to ionising radiation (refer to Fig. 3). As there has been no real attempt in the literature to directly characterise the response of TSPO after ionising radiation, TSPO is regarded as an attractive target for further studies and may delineate the complex pathways between neuroinflammation, ROS and antioxidant regulation, and the orchestration of a pro-inflammatory or anti-inflammatory responses after high or low dose irradiation. Because of the extreme sensitivity of TSPO expression to microglial activation, it is of interest to determine and profile the exact responses of microglia to ionising radiation at different doses, facilitated by TSPO. The remaining uncertainties surrounding neurobiological responses to ionising radiation necessitate further investigation, and the utility of a viable TSPO knockout mouse model, as well as the capacity for *in vivo* neuroimaging of TSPO using high affinity ligands, will facilitate new approaches to research in this field. Not only will this enhance understanding of the responses of the CNS to ionising radiation, it will also clarify the enigmatic role of TSPO in cell functioning.

## 6 Conclusion and open questions

The complex responses of the CNS under conditions of stress or pathology are largely underscored by neuroinflammation and mitochondrial redox balance. These mechanisms also coordinate responses to high and low dose ionising radiation. Exposure to high doses can impact the functionality of the CNS by inducing ROS formation, oxidative stress, alterations in DNA and gene expression, and activation of a neuroinflammatory response, all of which can propagate cellular damage. Whilst there is greater uncertainty surrounding responses to low dose irradiation, considerable evidence suggests that low dose exposure can manifest repair and protection to cells. Though there is sizeable literature challenging the current paradigm of the linear no-threshold model, the remaining contention and uncertainty surrounding low dose effects necessitates further studies which directly examine the mitochondrial mechanisms that stimulate antioxidant defences, as well as directly characterising the responses of microglia after low dose irradiation. This may also aid in investigating the dichotomous phenotypes of microglial activation, transitioning between M1/M2 activation states in response to low or high doses. Due to the ‘anti-inflammatory’ effects of low dose irradiation, it can be postulated that low dose ionising radiation may produce an M2 type microglial activation, which contributes to the reparative and protective effects that are present in many studies. This would create a ‘non-linear differential threshold’ of microglial activation in response to ionising radiation, transitioning between anti-inflammatory and pro-inflammatory functional states.

Collectively, animal radiobiological studies on the effects of high and low dose ionising radiation on the CNS highlight the variability in responses. It is most likely that responses are contingent not just on total dose, but dose rates, schedule of delivery and *in vitro/in vivo* experimental conditions. As mentioned prior, many studies focus on single dose irradiation schemes and measure acute cellular responses. In many cases, it has been reported that the outcomes of single dose exposure cannot predict or extend to outcomes after fractionated delivery, which produce disparate responses. Concomitantly, the effects of protracted/chronic dose delivery over a period time have different effects on cells, thus, further studies are needed in order to extrapolate radiation effects as a function of how doses are delivered. This may also be beneficial for studying the different responses of microglia to varying stimuli at varying doses and intensities, and may elucidate the cellular mechanisms that produce radioadaptive responses subsequent to priming with low dose irradiation.

In order to advance understanding of the mechanisms of radiation responses in the CNS, biomarkers of pathology may be of high utility moving forward in research. More comprehensive studies that exploit the exclusive upregulation of TSPO in activated microglia under conditions of stress in the brain will aid in explicating the role of neuroinflammatory responses after ionising radiation exposure, whilst also clarifying the exact function of TSPO and its role in cellular functioning. As more evidence emerges regarding the role of TSPO in modulating ROS, it may by extension lead to new insights into the role of TSPO in coordinating responses after ionising radiation that should be addressed in further studies. Furthermore, the controversial nature of low dose irradiation in conferring neuroprotection and the current lack of understanding of this phenomenon necessitate further investigation which will benefit from utilising TSPO, particularly its role in mitochondrial dependent processes. Future studies that elucidate the beneficial impact of low dose ionising radiation may ultimately uncover therapeutic potential in the prevention and treatment of sub-syndromal disorders associated with neuroinflammation, or may ameliorate the deleterious effects of neurodegenerative disorders. They may also pave the way for different approaches to setting radiation protection standards and regulatory bodies in making informed decisions, as well as a reassessment of the validity of the linear no-threshold model.

## Figures and Tables

**Table 1:** Chronology and selected major findings on the neurobiological impact of high dose ionising radiation.

**Year**	**IR type**	**Location**	**Total dose**	**Dose rate**	**Impact**	**Reference**
1896	X-radiation	–	–	–	X-rays and radioactivity are first described, X-rays are first utilised medically in cancer treatment	H. Becquerel [13] W.C. Röntgen [14]
1934	X-radiation	Cranial	–	–	Fractionated cancer treatments utilising X-radiation, effective in head and neck cancer	H. Coutard [12]
1964	Alpha particles	Cranial	60 Gy	10 Gy/min	Activation of microglia present in the cortex 48 h post-irradiation, degeneration of myelin. Suggested the peroxidation of lipids after irradiation	Estable-Puig et al. [15]
1994	X-ray	Cranial	10 Gy	0.2 Gy/min	Mn-SOD activity reduced by 30% in cerebral cortex	Yamaoka et al. [119]
2003	X-ray, single	Cranial	10 Gy	–	Neuroinflammation by activated microglia (CD68+/CD11b+) inhibits HC neurogenesis	Monje et al. [98]
2007	X-ray, single	Cranial	4 Gy	0.275 Gy/min	No microglial activation (Iba-1) and reversed proliferative capacity of HC progenitor cells after 1 week (Ki67)	Ben Abdallah et al. [97]
2012	γ-ray, single	In vitro	10 Gy	3.56 Gy/min	Alleviation of radiation-induced neuroinflammation via NF-κB and decreased ROS by application of PPARδ	Schnegg et al. [108]
	γ-ray, single dose	In vitro	10 Gy	3.7 Gy/min	MEK/ERK/1/2 signalling cascade activated c-Jun, increases ROS and pro-inflammatory cytokines via NADPH	Deng et al. [49]
	γ-ray, single	Cranial	2 Gy	1.079 Gy/min	Upregulation of apoptosis inducible genes *caspase-3* and *caspase-7*	Otani et al. [138]
2013	γ-ray, single ^56^Fe heavy ion, single	Whole body	2 Gy	1 Gy/min	Lipid peroxidation (MDA), oxidative stress, DNA DSBs, decreased DNA repair proteins, apoptosis and reduced cortical thickness	Suman et al. [114]
2015	γ-ray, single dose	Cranial	2, 3, 5 Gy	–	Reduced Cu/Zn/MnSOD in HC and cortex	Todorović et al. [80]
2016	γ-ray, single	Whole body	6 Gy	0.456 Gy/min	Increased DNA DSBs, lipid peroxidation (MDA), reduced GSH and SOD activity. Ameliorated with pre-treatment of antioxidant agent	Ismail et al. [113]

Abbreviations: Mn-SOD= Manganese superoxide dismutase, HC=hippocampus, ROS=reactive oxygen species, PPARδ=peroxisome proliferator-activated receptor delta, MDA=malondialdehyde, DSBs=double strand breaks, Cu/Zn-SOD= Copper/Zinc superoxide dismutase, GSH=glutathione

**Table 2:** Chronology and selected major findings on the neurobiological impact of low dose ionising radiation.

**Year**	**IR type**	**Location**	**Total dose**	**Dose rate**	**Impact**	**Reference**
1982	–	–	–	–	Benefits of low dose ionising radiation on health is formally proposed	T.D. Luckey [9]
1994	X-ray	Cranial	0.25, 0.5, 1 Gy	0.2 Gy/min	Mn-SOD activity increases by 20%. Lipid peroxides were reduced 15–20%	Yamaoka et al. [119]
1999	γ-ray, single	–	0.5 Gy	1.16 Gy/min	Increased GSH and CAT 3 h post-exposure, suppression of MPTP and lipid peroxidation (MDA) in PD model	Kojima et al. [128]
2003	γ-ray, single	Whole body	0.1 Gy	0.64 Gy/min	Neuroprotective changes in gene expression including stress response, synaptic signalling, DNA synthesis/repair, redox genes	Yin et al. [125]
2004	γ-ray, continuously for 5 weeks	Whole body	–	6x10^-6^ Gy/min, 2x10^-5^ Gy/min	In genetic autoimmune disease model, attenuated cerebral inflammation, increased lifespan	Ina et al. [135]
2008	γ-ray, 1 per week for 3 weeks	Whole body	0.5 Gy	0.88 Gy/min	Attenuated TNF-α, IL-6 in MS EAE model	Tsukimoto et al. [133]
2011	γ-ray, single	Whole body	0.02, 0.1, 0.5 Gy	0.81 Gy/min	Increased *SOD2* and SOD activity through NF-κB up to 8 days post-exposure (0.1 and 0.5 Gy)	Veeraraghavan et al. [143]
2012	γ-ray, single	Cranial	0.65 Gy	0.025 Gy/min	Decreased apoptosis, prevents further degeneration in retinitis pigmentosa model, up-regulation of *Prdx2* antioxidant gene	Otani et al. [138]
2014	γ-ray, single	Whole body	0.1 Gy, 1 Gy	2.07 Gy/min	Increased levels of GSH, catalase and SOD activity in brain tissue after 2 weeks, compared to the higher dose of 1 Gy	Tseng et al. [142]
2016	γ-ray, 2 fractions of 0.25 Gy	Whole body	0.5 Gy	0.45 Gy/min	Pre-exposure before insult reduces lipid peroxidation (MDA), apoptosis, increased GSH, SOD, CAT, improved cognitive function	Abdel-Rafei et al. [144]
	γ-ray, continuously for 72 h	In vitro	0.031 Gy, 0.124 Gy, 0.496 Gy	7.2x10^-6^ Gy/min, 2.9x10^-5^ Gy/min, 1.1x10^-4^ Gy/min	Dose-dependent alterations in apoptotic gene expression, inflammation, DNA DSBs, reduced cell survival and development	Katsura et al. [145]

Abbreviations: Mn-SOD= Manganese superoxide dismutase, GSH=glutathione, CAT=catalase, MPTP=(1-methyl-4-phenyl-1,2,3,6-tetrahydropyridine), MDA=malondialdehyde, PD=Parkinson's Disease, MS=Multiple Sclerosis, EAE=experimental autoimmune encephalomyelitis, ROS=reactive oxygen species, SOD=superoxide dismutase, DSBs=double strand breaks.

## References

[1] Harada K.H., Niisoe T., Imanaka M., Takahashi T., Amako K., Fujii Y., Kanameishi M., Ohse K., Nakai Y., Nishikawa T. (2014). Radiation dose rates now and in the future for residents neighboring restricted areas of the fukushima daiichi nuclear power plant. Proc. Natl. Acad. Sci. USA.

[2] Australian Radiation Protection and Nuclear Safety Agency, Ionising Radiation and Health, 2015.

[3] Seong K.M., Seo S., Lee D., Kim M.J., Lee S.S., Park S., Jin Y.W. (2016). Is the linear no-threshold dose-response paradigm still necessary for the assessment of health effects of low dose radiation?. J. Korean Med. Sci..

[4] ICRP, Low-dose extrapolation of radiation-related cancer risk. Icrp publication 99. Ann. ICRP, 2005, 35. 10.1016/j.icrp.2005.11.002 16782497

[5] Calabrese E.J. (2015). Historical foundations of hormesis. Homeopathy.

[6] Doss M. (2012). Evidence supporting radiation hormesis in atomic bomb survivor cancer mortality data. Dose Response.

[7] Doss M. (2014). Low dose radiation adaptive protection to control neurodegenerative diseases. Dose Response.

[8] Tang F.R., Loke W.K. (2015). Molecular mechanisms of low dose ionizing radiation-induced hormesis, adaptive responses, radioresistance, bystander effects, and genomic instability. Int J. Radiat. Biol..

[9] Luckey T.D. (1982). Physiological benefits from low levels of ionizing radiation. Health Phys..

[10] Gori T., Munzel T. (2012). Biological effects of low-dose radiation: Of harm and hormesis. Eur. Heart J..

[11] Calabrese E.J. (2015). Hormesis within a mechanistic context. Homeopathy.

[12] H. Coutard, Principles of x ray therapy of malignant diseases, The Lancet, 1934, 224, 1-4,e1,e2,5-8.

[13] Becquerel H. (1901). The radio-activity of matter. Nature.

[14] W.C. Röntgen, On a new kind or rays. Journal of the Franklin Institute, 141, 1896, pp. 183–191.

[15] Estable-Puig J.F., de Estable R.F., Tobias C., Haymaker W. (1964). Degeneration and regeneration of myelinated fibers in the cerebral and cerebellar cortex following damage from ionizing particle radiation. Acta Neuropathol..

[16] Nagai R., Tsunoda S., Hori Y., Asada H. (2000). Selective vulnerability to radiation in the hippocampal dentate granule cells. Surg. Neurol..

[17] Acharya M.M., Christie L.A., Hazel T.G., Johe K.K., Limoli C.L. (2014). Transplantation of human fetal-derived neural stem cells improves cognitive function following cranial irradiation. Cell Transplant..

[18] Pospisil P., Kazda T., Bulik M., Dobiaskova M., Burkon P., Hynkova L., Slampa P., Jancalek R. (2015). Hippocampal proton mr spectroscopy as a novel approach in the assessment of radiation injury and the correlation to neurocognitive function impairment: initial experiences. Radiat. Oncol..

[19] Parihar V.K., Limoli C.L. (2013). Cranial irradiation compromises neuronal architecture in the hippocampus. Proc. Natl. Acad. Sci. USA.

[20] Ye J., Jiang Z., Chen X., Liu M., Li J., Liu N. (2016). Electron transport chain inhibitors induce microglia activation through enhancing mitochondrial reactive oxygen species production. Exp. Cell Res..

[21] Limoli C.L., Giedzinski E., Rola R., Otsuka S., Palmer T.D., Fike J.R. (2004). Radiation response of neural precursor cells: Linking cellular sensitivity to cell cycle checkpoints, apoptosis and oxidative stress. Radiat. Res..

[22] Ma S., Kong B., Liu B., Liu X. (2013). Biological effects of low-dose radiation from computed tomography scanning. Int J. Radiat. Biol..

[23] Council N.R. (2006). Health Risks From Exposure to Low Levels of Ionizing Radiation: Beir vii - Phase 2.

[24] Morgan W.F., Bair W.J. (2013). Issues in low dose radiation biology: the controversy continues. A perspective. Radiat. Res..

[25] Su P., Zhang J., Wang D., Zhao F., Cao Z., Aschner M., Luo W. (2016). The role of autophagy in modulation of neuroinflammation in microglia. Neuroscience.

[26] Banati R.B., Egensperger R., Maassen A., Hager G., Kreutzberg G.W., Graeber M.B. (2004). Mitochondria in activated microglia in vitro. J. Neurocytol..

[27] Moneta M.E., Gehrmann J., Töpper R., Banati R.B., Kreutzberg G.W. (1993). Cell adhesion molecule expression in the regenerating rat facial nucleus. J. Neuroimmunol..

[28] Hurley S. (2003). Facial nerve axotomy in aged and young adult rats: analysis of the glial response. Neurobiol. Aging.

[29] Raivich G., Jones L.L., Kloss C.U.A., Werner A., Neumann H., Kreutzberg G.W. (1998). Immune surveillance in the injured nervous system: T-lymphocytes invade the axotomized mouse facial motor nucleus and aggregate around sites of neuronal degeneration. J. Neurosci..

[30] Gehrmann J., Banati R.B. (1995). Microglial turnover in the injured cns: Activated microglia undergo delayed DNA fragmentation following peripheral nerve injury. J. Neuropathol. Exp. Neurol..

[31] Banati R.B., Gehrmann J., Schubert P., Kreutzberg G.W. (1993). Cytotoxicity of microglia. Glia.

[32] Pathipati P., Muller S., Jiang X., Ferriero D. (2013). Phenotype and secretory responses to oxidative stress in microglia. Dev. Neurosci..

[33] Boche D., Perry V.H., Nicoll J.A. (2013). Review: Activation patterns of microglia and their identification in the human brain. Neuropathol. Appl. Neurobiol..

[34] Carreira B.P., Morte M.I., Santos A.I., Lourenco A.S., Ambrosio A.F., Carvalho C.M., Araujo I.M. (2014). Nitric oxide from inflammatory origin impairs neural stem cell proliferation by inhibiting epidermal growth factor receptor signaling. Front. Cell Neurosci..

[35] L'Episcopo F., Tirolo C., Testa N., Caniglia S., Morale M.C., Deleidi M., Serapide M.F., Pluchino S., Marchetti B. (2012). Plasticity of subventricular zone neuroprogenitors in mptp (1-methyl-4-phenyl-1,2,3,6-tetrahydropyridine) mouse model of parkinson's disease involves cross talk between inflammatory and wnt/beta-catenin signaling pathways: Functional consequences for neuroprotection and repair. J. Neurosci..

[36] Bisht K., Sharma K.P., Lecours C., Gabriela Sanchez M., El Hajj H., Milior G., Olmos-Alonso A., Gomez-Nicola D., Luheshi G., Vallieres L. (2016). Dark microglia: A new phenotype predominantly associated with pathological states. Glia.

[37] Wake H., Moorhouse A.J., Jinno S., Kohsaka S., Nabekura J. (2009). Resting microglia directly monitor the functional state of synapses in vivo and determine the fate of ischemic terminals. J. Neurosci..

[38] Di Filippo M., de Iure A., Giampa C., Chiasserini D., Tozzi A., Orvietani P.L., Ghiglieri V., Tantucci M., Durante V., Quiroga-Varela A. (2016). Persistent activation of microglia and nadph drive hippocampal dysfunction in experimental multiple sclerosis. Sci. Rep..

[39] Benedek G., Zhang J., Bodhankar S., Nguyen H., Kent G., Jordan K., Manning D., Vandenbark A.A., Offner H. (2016). Estrogen induces multiple regulatory b cell subtypes and promotes m2 microglia and neuroprotection during experimental autoimmune encephalomyelitis. J. Neuroimmunol..

[40] J. Wang, N. Song, H. Jiang, J. Wang, J. Xie, Pro-inflammatory cytokines modulate iron regulatory protein 1 expression and iron transportation through reactive oxygen/nitrogen species production in ventral mesencephalic neurons. Biochim Biophys Acta, 1832, 2013, pp. 618–625. 10.1016/j.bbadis.2013.01.021 23376588

[41] Haas S.J., Zhou X., Machado V., Wree A., Krieglstein K., Spittau B. (2016). Expression of tgfbeta1 and inflammatory markers in the 6-hydroxydopamine mouse model of parkinson's disease. Front. Mol. Neurosci..

[42] Tang Y., Le W. (2016). Differential roles of m1 and m2 microglia in neurodegenerative diseases. Mol. Neurobiol..

[43] Brandenburg S., Muller A., Turkowski K., Radev Y.T., Rot S., Schmidt C., Bungert A.D., Acker G., Schorr A., Hippe A. (2016). Resident microglia rather than peripheral macrophages promote vascularization in brain tumors and are source of alternative pro-angiogenic factors. Acta Neuropathol..

[44] Xu L., Nguyen J.V., Lehar M., Menon A., Rha E., Arena J., Ryu J., Marsh-Armstrong N., Marmarou C.R., Koliatsos V.E. (2016). Repetitive mild traumatic brain injury with impact acceleration in the mouse: Multifocal axonopathy, neuroinflammation, and neurodegeneration in the visual system. Exp. Neurol..

[45] Chanaday N.L., Roth G.A. (2016). Microglia and astrocyte activation in the frontal cortex of rats with experimental autoimmune encephalomyelitis. Neuroscience.

[46] Hu X., Li P., Guo Y., Wang H., Leak R.K., Chen S., Gao Y., Chen J. (2012). Microglia/macrophage polarization dynamics reveal novel mechanism of injury expansion after focal cerebral ischemia. Stroke.

[47] Miron V.E., Boyd A., Zhao J.W., Yuen T.J., Ruckh J.M., Shadrach J.L., van Wijngaarden P., Wagers A.J., Williams A., Franklin R.J. (2013). M2 microglia and macrophages drive oligodendrocyte differentiation during cns remyelination. Nat. Neurosci..

[48] Kigerl K.A., Gensel J.C., Ankeny D.P., Alexander J.K., Donnelly D.J., Popovich P.G. (2009). Identification of two distinct macrophage subsets with divergent effects causing either neurotoxicity or regeneration in the injured mouse spinal cord. J. Neurosci..

[49] Deng Z., Sui G., Rosa P.M., Zhao W. (2012). Radiation-induced c-jun activation depends on mek1-erk1/2 signaling pathway in microglial cells. PLoS One.

[50] Yin F., Boveris A., Cadenas E. (2014). Mitochondrial energy metabolism and redox signaling in brain aging and neurodegeneration. Antioxid. Redox Signal.

[51] Yuste J.E., Tarragon E., Campuzano C.M., Ros-Bernal F. (2015). Implications of glial nitric oxide in neurodegenerative diseases. Front Cell Neurosci..

[52] Liu P.W., Chen M.F., Tsai A.P., Lee T.J. (2012). Stat1 mediates oroxylin a inhibition of inos and pro-inflammatory cytokines expression in microglial bv-2 cells. PLoS One.

[53] Wang J.Y., Wen L.L., Huang Y.N., Chen Y.T., Ku M.C. (2006). Dual effects of antioxidants in neurodegeneration: Direct neuroprotection against oxidative stress and indirect protection via suppression of glia-mediated inflammation. Curr. Pharm. Des..

[54] T.R. Guilarte, M.K. Loth, S.R. Guariglia, Tspo finds nox2 in microglia for redox homeostasis, Trends Pharmacol. Sci., 2016 10.1016/j.tips.2016.02.008 PMC5042807 27113160

[55] Chen S.H., Oyarzabal E.A., Hong J.S. (2016). Critical role of the mac1/nox2 pathway in mediating reactive microgliosis-generated chronic neuroinflammation and progressive neurodegeneration. Curr. Opin. Pharm..

[56] J. Haslund-Vinding, G. McBean, V. Jaquet, F. Vilhardt, Nadph oxidases in microglia oxidant production: activating receptors, pharmacology, and association with disease, Br. J. Pharmacol. 2016. 10.1111/bph.13425 PMC5446574 26750203

[57] N. Sharma, B. Nehru, Apocyanin, a microglial nadph oxidase inhibitor prevents dopaminergic neuronal degeneration in lipopolysaccharide-induced parkinson's disease model. Mol. Neurobiol. 2015.. 10.1007/s12035-015-9267-2 26081143

[58] S. Yadav, S.K. Gandham, R. Panicucci, M.M. Amiji, Intranasal brain delivery of cationic nanoemulsion-encapsulated tnfalpha sirna in prevention of experimental neuroinflammation, Nanomedicine 2016 10.1016/j.nano.2015.12.374 PMC4837036 26767514

[59] Rojo A.I., McBean G., Cindric M., Egea J., Lopez M.G., Rada P., Zarkovic N., Cuadrado A. (2014). Redox control of microglial function: molecular mechanisms and functional significance. Antioxid. Redox Signal.

[60] Han J.E., Choi J.W. (2012). Control of jnk for an activation of nadph oxidase in lps-stimulated bv2 microglia. Arch. Pharm. Res..

[61] Levy Nogueira M., Epelbaum S., Steyaert J.M., Dubois B., Schwartz L. (2016). Mechanical stress models of alzheimer's disease pathology. Alzheimers Dement.

[62] Jiang T., Zhang Y.D., Chen Q., Gao Q., Zhu X.C., Zhou J.S., Shi J.Q., Lu H., Tan L., Yu J.T. (2016). Trem2 modifies microglial phenotype and provides neuroprotection in p301s tau transgenic mice. Neuropharmacology.

[63] Aquilano K., Baldelli S., Rotilio G., Ciriolo M.R. (2008). Role of nitric oxide synthases in parkinson's disease: A review on the antioxidant and anti-inflammatory activity of polyphenols. Neurochem. Res..

[64] Imai K., Kotani T., Tsuda H., Mano Y., Nakano T., Ushida T., Li H., Miki R., Sumigama S., Iwase A. (2016). Neuroprotective potential of molecular hydrogen against perinatal brain injury via suppression of activated microglia. Free Radic. Biol. Med..

[65] Boll M.C., Alcaraz-Zubeldia M., Montes S., Rios C. (2008). Free copper, ferroxidase and sod1 activities, lipid peroxidation and no(x) content in the csf. A different marker profile in four neurodegenerative diseases. Neurochem. Res..

[66] Onasanwo S.A., Velagapudi R., El-Bakoush A., Olajide O.A. (2016). Inhibition of neuroinflammation in bv2 microglia by the biflavonoid kolaviron is dependent on the nrf2/are antioxidant protective mechanism. Mol. Cell Biochem..

[67] Kam W.W., Banati R.B. (2013). Effects of ionizing radiation on mitochondria. Free Radic. Biol. Med..

[68] Einor D., Bonisoli-Alquati A., Costantini D., Mousseau T.A., Moller A.P. (2016). Ionizing radiation, antioxidant response and oxidative damage: a meta-analysis. Sci. Total Environ..

[69] Ji S., Tian Y., Lu Y., Sun R., Ji J., Zhang L., Duan S. (2014). Irradiation-induced hippocampal neurogenesis impairment is associated with epigenetic regulation of bdnf gene transcription. Brain Res..

[70] Osipov A.N., Buleeva G., Arkhangelskaya E., Klokov D. (2013). In vivo gamma-irradiation low dose threshold for suppression of DNA double strand breaks below the spontaneous level in mouse blood and spleen cells. Mutat. Res..

[71] Sweet T.B., Panda N., Hein A.M., Das S.L., Hurley S.D., Olschowka J.A., Williams J.P., O'Banion M.K. (2014). Central nervous system effects of whole-body proton irradiation. Radiat. Res..

[72] Roughton K., Bostrom M., Kalm M., Blomgren K. (2013). Irradiation to the young mouse brain impaired white matter growth more in females than in males. Cell Death Dis..

[73] Z. Ungvari, A. Podlutsky, D. Sosnowska, Z. Tucsek, P. Toth, F. Deak, T. Gautam, A. Csiszar, W.E. Sonntag, Ionizing radiation promotes the acquisition of a senescence-associated secretory phenotype and impairs angiogenic capacity in cerebromicrovascular endothelial cells: Role of increased DNA damage and decreased DNA repair capacity in microvascular radiosensitivity. J. Gerontol. A Biol. Sci. Med. Sci. 2013, 68, pp. 1443–1457. 10.1093/gerona/glt057 PMC3814240 23689827

[74] Huang T.T., Leu D., Zou Y. (2015). Oxidative stress and redox regulation on hippocampal-dependent cognitive functions. Arch. Biochem. Biophys..

[75] Sridharan D.M., Asaithamby A., Bailey S.M., Costes S.V., Doetsch P.W., Dynan W.S., Kronenberg A., Rithidech K.N., Saha J., Snijders A.M. (2015). Understanding cancer development processes after hze-particle exposure: Roles of ros, DNA damage repair and inflammation. Radiat. Res..

[76] Rana P., Khan A.R., Modi S., Hemanth Kumar B.S., Javed S., Tripathi R.P., Khushu S. (2013). Altered brain metabolism after whole body irradiation in mice: A preliminary in vivo 1h mrs study. Int J. Radiat. Biol..

[77] Jenrow K.A., Brown S.L., Lapanowski K., Naei H., Kolozsvary A., Kim J.H. (2013). Selective inhibition of microglia-mediated neuroinflammation mitigates radiation-induced cognitive impairment. Radiat. Res..

[78] Hua K., Schindler M.K., McQuail J.A., Forbes M.E., Riddle D.R. (2012). Regionally distinct responses of microglia and glial progenitor cells to whole brain irradiation in adult and aging rats. PLoS One.

[79] Rola R., Fishman K., Baure J., Rosi S., Lamborn K.R., Obenaus A., Nelson G.A., Fike J.R. (2008). Hippocampal neurogenesis and neuroinflammation after cranial irradiation with (56)fe particles. Radiat. Res..

[80] Todorovic A., Pejic S., Stojiljkovic V., Gavrilovic L., Popovic N., Pavlovic I., Saicic Z.S., Pajovic S.B. (2015). Antioxidative enzymes in irradiated rat brain-indicators of different regional radiosensitivity. Childs Nerv. Syst..

[81] Hekim N., Cetin Z., Nikitaki Z., Cort A., Saygili E.I. (2015). Radiation triggering immune response and inflammation. Cancer Lett..

[82] Osato K., Sato Y., Ochiishi T., Osato A., Zhu C., Sato M., Swanpalmer J., Modjtahedi N., Kroemer G., Kuhn H.G. (2010). Apoptosis-inducing factor deficiency decreases the proliferation rate and protects the subventricular zone against ionizing radiation. Cell Death Dis..

[83] Marples B., Wouters B.G., Joiner M.C. (2003). An association between the radiation-induced arrest of g2-phase cells and low-dose hyper-radiosensitivity: a plausible underlying mechanism?. Radiat. Res..

[84] Mizumatsu S., Monje M.L., Morhardt D.R., Rola R., Palmer T.D., Fike J.R. (2003). Extreme sensitivity of adult neurogenesis to low doses of x-irradiation. Cancer Res..

[85] Monje M., Thomason M.E., Rigolo L., Wang Y., Waber D.P., Sallan S.E., Golby A.J. (2013). Functional and structural differences in the hippocampus associated with memory deficits in adult survivors of acute lymphoblastic leukemia. Pedia. Blood Cancer.

[86] Zakhvataev V.E. (2015). Possible scenarios of the influence of low-dose ionizing radiation on neural functioning. Med. Hypotheses.

[87] Raber J., Rola R., LeFevour A., Morhardt D., Curley J., Mizumatsu S., VandenBerg S.R., Fike J.R. (2004). Radiation-induced cognitive impairments are associated with changes in indicators of hippocampal neurogenesis. Radiat. Res..

[88] Acharya M.M., Lan M.L., Kan V.H., Patel N.H., Giedzinski E., Tseng B.P., Limoli C.L. (2010). Consequences of ionizing radiation-induced damage in human neural stem cells. Free Radic. Biol. Med..

[89] Saeed Y., Xie B., Xu J., Wang H., Hassan M., Wang R., Hong M., Hong Q., Deng Y. (2014). Indirect effects of radiation induce apoptosis and neuroinflammation in neuronal sh-sy5y cells. Neurochem. Res..

[90] Hong J.H., Chiang C.S., Campbell I.L., Sun J.R., Withers H.R., McBride W.H. (1995). Induction of acute-phase gene-expression by brain irradiation. Int. J. Radiat. Oncol. Biol. Phys..

[91] Hwang S.Y., Jung J.S., Kim T.H., Lim S.J., Oh E.S., Kim J.Y., Ji K.A., Joe E.H., Cho K.H., Han I.O. (2006). Ionizing radiation induces astrocyte gliosis through microglia activation. Neurobiol. Dis..

[92] Raber J., Allen A.R., Sharma S., Allen B., Rosi S., Olsen R.H., Davis M.J., Eiwaz M., Fike J.R., Nelson G.A. (2016). Effects of proton and combined proton and (56)fe radiation on the hippocampus. Radiat. Res..

[93] Bartsch T., Wulff P. (2015). The hippocampus in aging and disease: From plasticity to vulnerability. Neuroscience.

[94] Conner K.R., Payne V.S., Forbes M.E., Robbins M.E., Riddle D.R. (2010). Effects of the at1 receptor antagonist l-158,809 on microglia and neurogenesis after fractionated whole-brain irradiation. Radiat. Res..

[95] Rola R., Otsuka S., Obenaus A., Nelson G.A., Limoli C.L., VandenBerg S.R., Fike J.R. (2004). Indicators of hippocampal neurogenesis are altered by 56fe- particle irradiation in a dose-dependent manner. Radiat. Res..

[96] M.L. Monje, H. Vogel, M. Masek, K.L. Ligon, P.G. Fisher, T.D. Palmer, Impaired human hippocampal neurogenesis after treatment for central nervous system malignancies. Ann. Neurol. 2007, vol. 62, pp. 515–520 10.1002/ana.21214 17786983

[97] Ben Abdallah N.M., Slomianka L., Lipp H.P. (2007). Reversible effect of x-irradiation on proliferation, neurogenesis, and cell death in the dentate gyrus of adult mice. Hippocampus.

[98] Monje M.L., Toda H., Palmer T.D. (2003). Inflammatory blockade restores adult hippocampal neurogenesis. Science.

[99] Morganti J.M., Jopson T.D., Liu S., Gupta N., Rosi S. (2014). Cranial irradiation alters the brain's microenvironment and permits ccr2+ macrophage infiltration. PLoS One.

[100] Acharya M.M., Patel N.H., Craver B.M., Tran K.K., Giedzinski E., Tseng B.P., Parihar V.K., Limoli C.L. (2015). Consequences of low dose ionizing radiation exposure on the hippocampal microenvironment. PLoS One.

[101] Kalm M., Fukuda A., Fukuda H., Ohrfelt A., Lannering B., Bjork-Eriksson T., Blennow K., Marky I., Blomgren K. (2009). Transient inflammation in neurogenic regions after irradiation of the developing brain. Radiat. Res..

[102] Li M.D., Burns T.C., Kumar S., Morgan A.A., Sloan S.A., Palmer T.D. (2015). Aging-like changes in the transcriptome of irradiated microglia. Glia.

[103] Davella D., Cicciarello R., Albiero F., Mesiti M., Gagliardi M.E., Russi E., Daquino A., Tomasello F., Daquino S. (1992). Quantitative study of blood-brain-barrier permeability changes after experimental whole-brain radiation. Neurosurgery.

[104] Li Y.Q., Chen P., Haimovitz-Friedman A., Reilly R.M., Wong C.S. (2003). Endothelial apoptosis initiates acute blood-brain barrier disruption after ionizing radiation. Cancer Res..

[105] Pena L.A., Fuks Z., Kolesnick R.N. (2000). Radiation-induced apoptosis of endothelial cells in the murine central nervous system: Protection by fibroblast growth factor and sphingomyelinase deficiency. Cancer Res..

[106] Gaber M.W., Yuan H., Killmar J.T., Naimark M.D., Kiani M.F., Merchant T.E. (2004). An intravital microscopy study of radiation-induced changes in permeability and leukocyte-endothelial cell interactions in the microvessels of the rat pia mater and cremaster muscle. Brain Res. Protoc..

[107] Lee W.H., Sonntag W.E., Mitschelen M., Yan H., Lee Y.W. (2010). Irradiation induces regionally specific alterations in pro-inflammatory environments in rat brain. Intl J. Radiat. Biol..

[108] Schnegg C.I., Kooshki M., Hsu F.C., Sui G., Robbins M.E. (2012). Ppardelta prevents radiation-induced proinflammatory responses in microglia via transrepression of nf-kappab and inhibition of the pkcalpha/mek1/2/erk1/2/ap-1 pathway. Free Radic. Biol. Med..

[109] Dong X., Luo M., Huang G., Zhang J., Tong F., Cheng Y., Cai Q., Dong J., Wu G., Cheng J. (2015). Relationship between irradiation-induced neuro-inflammatory environments and impaired cognitive function in the developing brain of mice. Int J. Radiat. Biol..

[110] Ramanan S., Kooshki M., Zhao W., Hsu F.C., Robbins M.E. (2008). Pparalpha ligands inhibit radiation-induced microglial inflammatory responses by negatively regulating nf-kappab and ap-1 pathways. Free Radic. Biol. Med..

[111] R. Rola, Y. Zou, T.T. Huang, K. Fishman, J. Baure, S. Rosi, H. Milliken, C.L. Limoli, J.R. Fike, Lack of extracellular superoxide dismutase (ec-sod) in the microenvironment impacts radiation-induced changes in neurogenesis. Free Radic. Biol. Med. 2007, vol. 42, pp. 1133–1145; discussion 1131–1132 10.1016/j.freeradbiomed.2007.01.020 PMC1934512 17382195

[112] Leach J.K., Van Tuyle G., Lin P.S., Schmidt-Ullrich R., Mikkelsen R.B. (2001). Ionizing radiation-induced, mitochondria-dependent generation of reactive oxygen/nitrogen. Cancer Res..

[113] Ismail A.F., El-Sonbaty S.M. (2016). Fermentation enhances Ginkgo biloba protective role on gamma-irradiation induced neuroinflammatory gene expression and stress hormones in rat brain. J. Photochem. Photobiol. B.

[114] Suman S., Rodriguez O.C., Winters T.A., Fornace A.J., Albanese C., Datta K. (2013). Therapeutic and space radiation exposure of mouse brain causes impaired dna repair response and premature senescence by chronic oxidant production. Aging.

[115] Fishman K., Baure J., Zou Y., Huang T.T., Andres-Mach M., Rola R., Suarez T., Acharya M., Limoli C.L., Lamborn K.R. (2009). Radiation-induced reductions in neurogenesis are ameliorated in mice deficient in cuznsod or mnsod. Free Radic. Biol. Med..

[116] Liao A.C., Craver B.M., Tseng B.P., Tran K.K., Parihar V.K., Acharya M.M., Limoli C.L. (2013). Mitochondrial-targeted human catalase affords neuroprotection from proton irradiation. Radiat. Res..

[117] Parihar V.K., Allen B.D., Tran K.K., Chmielewski N.N., Craver B.M., Martirosian V., Morganti J.M., Rosi S., Vlkolinsky R., Acharya M.M. (2015). Targeted overexpression of mitochondrial catalase prevents radiation-induced cognitive dysfunction. Antioxid. Redox Signal.

[118] Greene-Schloesser D.M., Kooshki M., Payne V., D'Agostino R.B., Wheeler K.T., Metheny-Barlow L.J., Robbins M.E. (2014). Cellular response of the rat brain to single doses of (137)cs gamma rays does not predict its response to prolonged 'biologically equivalent' fractionated doses. Int J. Radiat. Biol..

[119] Yamaoka K., Edamatsu R., Itoh T., Akitane M. (1994). Effects of low-dose x-ray irradiation on biomembrane in brain cortex of aged rats. Free Radic. Biol. Med..

[120] Wang B., Tanaka K., Ji B., Ono M., Fang Y., Ninomiya Y., Maruyama K., Izumi-Nakajima N., Begum N., Higuchi M. (2014). Total body 100-mgy x-irradiation does not induce alzheimer's disease-like pathogenesis or memory impairment in mice. J. Radiat. Res..

[121] L. Chien, W.K. Chen, S.T. Liu, C.R. Chang, M.C. Kao, K.W. Chen, S.C. Chiu, M.L. Hsu, I.C. Hsiang, Y.J. Chen, et al., Low-dose ionizing radiation induces mitochondrial fusion and increases expression of mitochondrial complexes i and iii in hippocampal neurons. Oncotarget 2015, vol. 6, pp. 30628–30639. 10.18632/oncotarget.5790 PMC4741557 26415228

[122] Baulch J.E., Craver B.M., Tran K.K., Yu L., Chmielewski N., Allen B.D., Limoli C.L. (2015). Persistent oxidative stress in human neural stem cells exposed to low fluences of charged particles. Redox Biol..

[123] Otsuka K., Koana T., Tauchi H., Sakai K. (2006). Activation of antioxidative enzymes induced by low-dose-rate whole-body gamma irradiation: Adaptive response in terms of initial DNA damage. Radiat. Res..

[124] Day T.K., Zeng G., Hooker A.M., Bhat M., Scott B.R., Turner D.R., Sykes P.J. (2007). Adaptive response for chromosomal inversions in pkz1 mouse prostate induced by low doses of x radiation delivered after a high dose. Radiat. Res..

[125] Yin E., Nelson D.O., Coleman M.A., Peterson L.E., Wyrobek A.J. (2003). Gene expression changes in mouse brain after exposure to low-dose ionizing radiation. Int J. Radiat. Biol..

[126] Lowe X.R., Bhattacharya S., Marchetti F., Wyrobek A.J. (2009). Early brain response to low-dose radiation exposure involves molecular networks and pathways associated with cognitive functions, advanced aging and alzheimer's disease. Radiat. Res..

[127] Silasi G., Diaz-Heijtz R., Besplug J., Rodriguez-Juarez R., Titov V., Kolb B., Kovalchuk O. (2004). Selective brain responses to acute and chronic low-dose x-ray irradiation in males and females. Biochem. Biophys. Res. Commun..

[128] Kojima S., Matsuki O., Nomura T., Yamaoka K., Takahashi M., Niki E. (1999). Elevation of antioxidant potency in the brain of mice by low-dose γ- ray irradiation and its effect on 1-methyl-4-phenyl-1,2,3,6- tetrahydropyridine (mptp)-induced brain damage. Free Radic. Biol. Med..

[129] El-Ghazaly M.A., Sadik N.A., Rashed E.R., Abd-El-Fattah A.A. (2015). Neuroprotective effect of egb761(r) and low-dose whole-body gamma-irradiation in a rat model of parkinson's disease. Toxicol. Ind. Health.

[130] Peterson K., Rosenblum M.K., Powers J.M., Alvord E., Walker R.W., Posner J.B. (1993). Effect of brain irradiation on demyelinating lesions. Neurology.

[131] Cook S.D., Troiano R., Zito G., Lavenhar M., Devereux C., Hafstein M.P., Hernandez E., Vidaver R., Dowling P.C. (1986). Effect of total lymphoid irradiation in chronic progressive multiple-sclerosis. Lancet.

[132] C. Devereux, R. Troiano, G. Zito, R.B. Devereux, K.J. Kopecky, R. Friedman, P.C. Dowling, M.P. Hafstein, C. Rohowskykochan, S.D. Cook, Effect of total lymphoid irradiation on functional status in chronic multiple-sclerosis - importance of lymphopenia early after treatment – the pros. Neurology 1988, vol. 38, pp. 32–37. 3290713

[133] Tsukimoto M., Nakatsukasa H., Sugawara K., Yamashita K., Kojima S. (2008). Repeated 0.5-gy γ irradiation attenuates experimental autoimmune encephalomyelitis with up-regulation of regulatory t cells and suppression of il17 production. Radiat. Res..

[134] Shimura N., Kojima S. (2014). Effects of low-dose-gamma rays on the immune system of different animal models of disease. Dose Response.

[135] Ina Y., Sakai K. (2004). Prolongation of life span associated with immunological modification by chronic low-dose-rate irradiation in mrl-lpr/lprmice. Radiat. Res..

[136] Kataoka T., Etani R., Takata Y., Nishiyama Y., Kawabe A., Kumashiro M., Taguchi T., Yamaoka K. (2014). Radon inhalation protects against transient global cerebral ischemic injury in gerbils. Inflammation.

[137] Yoshimoto M., Kataoka T., Toyota T., Taguchi T., Yamaoka K. (2012). Inhibitory effects of prior low-dose x-irradiation on cold-induced brain injury in mouse. Inflammation.

[138] Otani A., Kojima H., Guo C., Oishi A., Yoshimura N. (2012). Low-dose-rate, low-dose irradiation delays neurodegeneration in a model of retinitis pigmentosa. Am. J. Pathol..

[139] Cobbs C.S., Levi D.S., Aldape K., Israel M.A. (1996). Manganese superoxide dismutase expression in human central nervous system tumors. Cancer Res..

[140] Russo G.L., Tedesco I., Russo M., Cioppa A., Andreassi M.G., Picano E. (2012). Cellular adaptive response to chronic radiation exposure in interventional cardiologists. Eur. Heart J..

[141] Eken A., Aydin A., Erdem O., Akay C., Sayal A., Somuncu I. (2012). Induced antioxidant activity in hospital staff occupationally exposed to ionizing radiation. Int J. Radiat. Biol..

[142] Tseng B.P., Giedzinski E., Izadi A., Suarez T., Lan M.L., Tran K.K., Acharya M.M., Nelson G.A., Raber J., Parihar V.K. (2014). Functional consequences of radiation-induced oxidative stress in cultured neural stem cells and the brain exposed to charged particle irradiation. Antioxid. Redox Signal.

[143] Veeraraghavan J., Natarajan M., Herman T.S., Aravindan N. (2011). Low-dose gamma-radiation-induced oxidative stress response in mouse brain and gut: Regulation by nfkappab-mnsod cross-signaling. Mutat. Res..

[144] M .Abdel-Rafei, M. Amin, H. Hasan, Novel effect of daflon and low-dose -radiation in modulation of thioacetamide-induced hepatic encephalopathy in male albino rats. Human & Experimental Toxicology 2016. 10.1177/0960327116637657 26987350

[145] Katsura M., Cyou-Nakamine H., Zen Q., Zen Y., Nansai H., Amagasa S., Kanki Y., Inoue T., Kaneki K., Taguchi A. (2016). Effects of chronic low-dose radiation on human neural progenitor cells. Sci. Rep..

[146] Banati R.B. (2002). Visualising microglial activation in vivo. GLIA.

[147] Banati R.B. (2003). Neuropathological imaging: In vivo detection of glial activation as a measure of disease and adaptive change in the brain. Br. Med. Bull..

[148] B. Janssen, D.J. Vugts, U. Funke, G.T. Molenaar, P.S. Kruijer, B.N. van Berckel, A.A. Lammertsma, A.D. Windhorst, Imaging of neuroinflammation in alzheimer's disease, multiple sclerosis and stroke: Recent developments in positron emission tomography. Biochim. Biophys. Acta, vol. 1862, 2016, pp. 425–441. 10.1016/j.bbadis.2015.11.011 26643549

[149] Ching A.S., Kuhnast B., Damont A., Roeda D., Tavitian B., Dolle F. (2012). Current paradigm of the 18-kda translocator protein (tspo) as a molecular target for pet imaging in neuroinflammation and neurodegenerative diseases. Insights Imaging.

[150] Fan J., Campioli E., Midzak A., Culty M., Papadopoulos V. (2015). Conditional steroidogenic cell-targeted deletion of tspo unveils a crucial role in viability and hormone-dependent steroid formation. Proc. Natl. Acad. Sci. USA.

[151] Banati R.B., Middleton R.J., Chan R., Hatty C.R., Kam W.W., Quin C., Graeber M.B., Parmar A., Zahra D., Callaghan P. (2014). Positron emission tomography and functional characterization of a complete pbr/tspo knockout. Nat. Commun..

[152] Middleton R.J., Liu G.J., Banati R.B. (2015). Guwiyang wurra--'fire mouse': A global gene knockout model for tspo/pbr drug development, loss-of-function and mechanisms of compensation studies. Biochem. Soc. Trans..

[153] Wang M., Wang X., Zhao L., Ma W., Rodriguez I.R., Fariss R.N., Wong W.T. (2014). Macroglia-microglia interactions via tspo signaling regulates microglial activation in the mouse retina. J. Neurosci..

[154] Banati R.B., Goerres G.W., Myers R., Gunn R.N., Turkheimer F.E., Kreutzberg G.W., Brooks D.J., Jones T., Duncan J.S. (1999). [11c] (r)-pk11195 positron emission tomography imaging of activated microglia in vivo in rasmussen's encephalitis. Neurology.

[155] Pappata S., Levasseur M., Gunn R.N., Myers R., Crouzel C., Syrota A., Jones T., Kreutzberg G.W., Banati R.B. (2000). Thalamic microglial activation in ischemic stroke detected in vivo by pet and [(11)c]pk11195. Neurology.

[156] Cagnin A., Kassiou M., Meikle S.R., Banati R.B. (2006). In vivo evidence for microglial activation in neurodegenerative dementia. Acta Neurol. Scand..

[157] Cagnin A., Gerhard A., Banati R.B. (2002). In vivo imaging of neuroinflammation. Eur. Neuropsychopharmacol..

[158] Banati R.B., Newcombe J., Gunn R.N., Cagnin A., Turkheimer F., Heppner F., Price G., Wegner F., Giovannoni G., Miller D.H. (2000). The peripheral benzodiazepine binding site in the brain in multiple sclerosis. Quantitative in vivo imaging of microglia as a measure of disease activity. Brain.

[159] Edison P., Archer H.A., Gerhard A., Hinz R., Pavese N., Turkheimer F.E., Hammers A., Tai Y.F., Fox N., Kennedy A. (2008). Microglia, amyloid, and cognition in alzheimer's disease: an [11c](r)pk11195-pet and [11c]pib-pet study. Neurobiol. Dis..

[160] Gerhard A., Pavese N., Hotton G., Turkheimer F., Es M., Hammers A., Eggert K., Oertel W., Banati R.B., Brooks D.J. (2006). In vivo imaging of microglial activation with [11c](r)-pk11195 pet in idiopathic parkinson's disease. Neurobiol. Dis..

[161] Loth M.K., Choi J., McGlothan J.L., Pletnikov M.V., Pomper M.G., Guilarte T.R. (2016). Tspo in a murine model of sandhoff disease: Presymptomatic marker of neurodegeneration and disease pathophysiology. Neurobiol. Dis..

[162] Varrone A., Oikonen V., Forsberg A., Joutsa J., Takano A., Solin O., Haaparanta-Solin M., Nag S., Nakao R., Al-Tawil N. (2015). Positron emission tomography imaging of the 18-kda translocator protein (tspo) with [18f]fempa in alzheimer's disease patients and control subjects. Eur. J. Nucl. Med. Mol. Imaging.

[163] A. Cagnin, M. Rossor, E.L. Sampson, T. Mackinnon, R.B. Banati, In vivo detection of microglial activation in frontotemporal dementia. Ann. Neurol. vol. 56, 2004, pp. 894–897. 10.1002/ana.20332 15562429

[164] Zurcher N.R., Loggia M.L., Lawson R., Chonde D.B., Izquierdo-Garcia D., Yasek J.E., Akeju O., Catana C., Rosen B.R., Cudkowicz M.E. (2015). Increased in vivo glial activation in patients with amyotrophic lateral sclerosis: Assessed with [(11)c]-pbr28. Neuroimage Clin..

[165] Turner M.R., Cagnin A., Turkheimer F.E., Miller C.C.J., Shaw C.E., Brooks D.J., Leigh P.N., Banati R.B. (2004). Evidence of widespread cerebral microglial activation in amyotrophic lateral sclerosis: An [11c](r)-pk11195 positron emission tomography study. Neurobiol. Dis..

[166] Rissanen E., Tuisku J., Rokka J., Paavilainen T., Parkkola R., Rinne J.O., Airas L. (2014). In vivo detection of diffuse inflammation in secondary progressive multiple sclerosis using pet imaging and the radioligand (1)(1)c-pk11195. J. Nucl. Med..

[167] Corcia P., Tauber C., Vercoullie J., Arlicot N., Prunier C., Praline J., Nicolas G., Venel Y., Hommet C., Baulieu J.L. (2012). Molecular imaging of microglial activation in amyotrophic lateral sclerosis. PLoS One.

[168] Tsartsalis S., Dumas N., Tournier B.B., Pham T., Moulin-Sallanon M., Gregoire M.C., Charnay Y., Millet P. (2015). Spect imaging of glioma with radioiodinated clinde: Evidence from a mouse gl26 glioma model. EJNMMI Res..

[169] Werry E.L., Barron M.L., Kassiou M. (2015). Tspo as a target for glioblastoma therapeutics. Biochem. Soc. Trans..

[170] Ma L., Zhang H., Liu N., Wang P.Q., Guo W.Z., Fu Q., Jiao L.B., Ma Y.Q., Mi W.D. (2016). Tspo ligand pk11195 alleviates neuroinflammation and beta-amyloid generation induced by systemic lps administration. Brain Res. Bull..

[171] Gut P., Baeza-Raja B., Andersson O., Hasenkamp L., Hsiao J., Hesselson D., Akassoglou K., Verdin E., Hirschey M.D., Stainier D.Y. (2013). Whole-organism screening for gluconeogenesis identifies activators of fasting metabolism. Nat. Chem. Biol..

[172] Scholz R., Caramoy A., Bhuckory M.B., Rashid K., Chen M., Xu H., Grimm C., Langmann T. (2015). Targeting translocator protein (18 kda) (tspo) dampens pro-inflammatory microglia reactivity in the retina and protects from degeneration. J. Neuroinflamm..

[173] Rupprecht R., Papadopoulos V., Rammes G., Baghai T.C., Fan J., Akula N., Groyer G., Adams D., Schumacher M. (2010). Translocator protein (18 kda) (tspo) as a therapeutic target for neurological and psychiatric disorders. Nat. Rev. Drug Disco..

[174] Zhao A.H., Tu L.N., Mukai C., Sirivelu M.P., Pillai V.V., Morohaku K., Cohen R., Selvaraj V. (2016). Mitochondrial translocator protein (tspo) function is not essential for heme biosynthesis. J. Biol. Chem..

[175] Gatliff J., Campanella M. (2012). The 18 kda translocator protein (tspo): a new perspective in mitochondrial biology. Curr. Mol. Med..

[176] L. Veenman, J. Alten, K. Linnemannstons, Y. Shandalov, S. Zeno, M. Lakomek, M. Gavish, W. Kugler, Potential involvement of f0f1-atp(synth)ase and reactive oxygen species in apoptosis induction by the antineoplastic agent erucylphosphohomocholine in glioblastoma cell lines : A mechanism for induction of apoptosis via the 18 kda mitochondrial translocator protein. Apoptosis 2010, 15, pp. 753–768. 10.1007/s10495-010-0460-5 PMC3128697 20107899

[177] Veenman L., Gavish M. (2012). The role of 18 kda mitochondrial translocator protein (tspo) in programmed cell death, and effects of steroids on tspo expression. Curr. Mol. Med..

[178] Gatliff J., East D., Crosby J., Abeti R., Harvey R., Craigen W., Parker P., Campanella M. (2014). Tspo interacts with vdac1 and triggers a ros-mediated inhibition of mitochondrial quality control. Autophagy.

[179] Gatliff J., Campanella M. (2015). Tspo is a redox regulator of cell mitophagy. Biochem. Soc. Trans..

[180] Lin R., Angelin A., Da Settimo F., Martini C., Taliani S., Zhu S., Wallace D.C. (2014). Genetic analysis of dtspo, an outer mitochondrial membrane protein, reveals its functions in apoptosis, longevity, and aβ42-induced neurodegeneration. Aging Cell.

[181] Zeno S., Veenman L., Katz Y., Bode J., Gavish M., Zaaroor M. (2012). The 18 kda mitochondrial translocator protein (tspo) prevents accumulation of protoporphyrin ix. Involvement of reactive oxygen species (ros). Curr. Mol. Med..

[182] Shargorodsky L., Veenman L., Caballero B., Pe'er Y., Leschiner S., Bode J., Gavish M. (2012). The nitric oxide donor sodium nitroprusside requires the 18 kda translocator protein to induce cell death. Apoptosis.

[183] Santoro A., Mattace Raso G., Taliani S., Da Pozzo E., Simorini F., Costa B., Martini C., Laneri S., Sacchi A., Cosimelli B. (2016). Tspo-ligands prevent oxidative damage and inflammatory response in c6 glioma cells by neurosteroid synthesis. Eur. J. Pharm. Sci..

[184] Choi J., Ifuku M., Noda M., Guilarte T.R. (2011). Translocator protein (18 kda)/peripheral benzodiazepine receptor specific ligands induce microglia functions consistent with an activated state. Glia.

[185] Batarseh A., Li J., Papadopoulos V. (2010). Protein kinase cε regulation of translocator protein (18 kda) tspo gene expression is mediated through a mapk pathway targeting stat3 and c-jun transcription factors. Biochemistry.

